# RNA-Seq Analysis of the Effect of Zinc Deficiency on *Microsporum canis*, *ZafA* Gene Is Important for Growth and Pathogenicity

**DOI:** 10.3389/fcimb.2021.727665

**Published:** 2021-09-16

**Authors:** Pengxiu Dai, Yangou Lv, Xiaowen Gong, Jianye Han, Peng Gao, Haojie Xu, Yihua Zhang, Xinke Zhang

**Affiliations:** ^1^The College of Veterinary Medicine, Northwest Agriculture and Forestry University, Yangling, China; ^2^The Animal Health Supervision Institute of Xi’an, Xi’an, China; ^3^The Animal Health Supervision Institute of Yanta, Xi’an, China

**Keywords:** *Microsporum canis*, zinc deficiency, RNA-Seq, ATMT, functional verification, *ZafA*

## Abstract

*Microsporum canis*, a common pathogenic skin fungus, can cause dermatophytosis in humans and animals. Zinc is an important trace element and plays an important role in the growth and metabolism of fungi. Currently, the effects of zinc deficiency on growth, gene expression, and metabolic pathway have not been clarified in *M. canis*. Therefore, *M. canis* was cultured under zinc restriction, and RNA-Seq was conducted in this study. The growth of *M. canis* was severely inhibited, and many genes showed significant upregulation and downregulation in *M. canis* with zinc deficiency. Zinc deficiency could negatively affect the gene expression and biological metabolic pathway in *M. canis*. The zinc-responsiveness transcriptional activator (*ZafA*) gene was significantly upregulated and shared homology with *Zap1*. Thus, the *ZafA* gene might be the main transcription factor regulating *M. canis* zinc homeostasis. The *ZafA* gene knockout strain, ZafA-hph, was constructed *via Agrobacterium tumefaciens*-mediated transformation (ATMT) in *M. canis* for the first time to assess its function. *In vitro* growth ability, hair biodegradation ability, virulence test, and zinc absorption capacity in ZafA-hph and wild-type *M. canis* strains were compared. Results showed that the *ZafA* gene plays an important role in zinc absorption, expression of zinc transporter genes, and growth and pathogenicity in *M. canis* and can be used as a new drug target. Cutting off the zinc absorption pathway can be used as a way to prevent and control infection in *M. canis*.

## Introduction

Dermatomycosis is a common disease in humans and fur animals, and its occurrence is closely related to the animal’s internal and external factors, such as life environment or autoimmune status. In the past, the main targets of dermatomycosis are fur animals raised in high-density settings, and such infections in humans and other animals are mostly sporadic. In recent years, with increasing number of families raising and/or living with companion animals, such as dogs and cats, the close contact between humans and fur animals has increased the incidence of skin mycosis (Nenoff et al., 2012). Zoonotic dermatomycosis has become the main pathogenic factor linked with fungal skin diseases in human and other animals (Nakamura et al., 1999; Stojanov et al., 2009). As the causative agent for the highest incidence of zoonotic dermatomycosis, *M. canis* is widely prevalent worldwide (Broshnissimov et al., 2018; Takeda et al., 2018). The superficial mycosis caused by *M. canis* has many adverse effects on human health and fur animals. However, the poor adaptability of genetic manipulation and insufficient understanding of the disease mean that studies on *M. canis* have focused on drug resistance and animal tests for this pathogen (Achterman and White, 2012). Therefore, *M. canis* should be further studied.

Zinc is an essential microelement in organisms and plays an important role in cells. Zinc is a structural and catalytic cofactor for many proteins (Satoru et al., 2007). Approximately 8% of proteins are thought to need zinc binding for functionality in *Saccharomyces cerevisiae* (North et al., 2012). Fungi have evolved a complex system for regulating zinc absorption and transport to maintain the proper concentration of zinc ions in cells and ensure their normal functioning (Wilson et al., 2012; Silva-Bailão et al., 2017). Zinc ion absorption in *S. cerevisiae* is regulated by Zap1 (Eide, 1998). Genes sharing homology with *Zap1* have also been identified in pathogenic fungi such as *Aspergillus fumigatus*, *Candida albicans*, and *Cryptococcus gattii* (Moreno et al., 2007; Kim et al., 2008; Schneider Rde et al., 2012; Jung, 2015).

However, the effects of zinc deficiency on gene expression, metabolic pathways, growth, and pathogenicity have not been clarified in *M. canis*. Therefore, in the present study, we have performed the RNA-Seq of *M. canis* in the absence of zinc. The *ZafA* gene, which shares homology with *Zap1*, is significantly upregulated in *M. canis* grown under zinc restriction and may be the main transcription factor regulating *M. canis* zinc homeostasis. Thus, the function of the *ZafA* gene is assessed. Results lead to future work where the family of genes related to zinc absorption is further explored systematically.

## Materials and Methods

### Strains and Media

The wild-type CBS 113480 *M. canis* strain was cultured on Sabouraud Dextrose Agar (SDA) at 28°C for 14 days (Shi et al., 2016). Sterile sodium chloride solution (5 ml, 0.9%) was added into the SDA plate. The culture plate was wrapped with Parafilm and oscillated on a vortex oscillator for several seconds. The fungal conidia suspension was collected, and the cell counting plate was used for counting. Concentrations were adjusted to 10^8^ CFU/ml.

Chelex-treated SDA (SDA-Zn-1) was used to limit zinc availability (Lyons et al., 2000; Cho et al., 2007). Twenty-five grams of the Chelex-100 ion exchange resin (25 g, Sigma, USA) was added into 1 l liquid SDA (no agar), and the mixture was stirred overnight at 4°C (pH > 4). After the removal of resin, MnSO_4_, FeCl_3_, CuSO_4_, CaCl_2_, MgSO_4_, and KPO_4_ monobasic were added to obtain recommended (Bio101) final concentrations. Atomic absorption spectroscopy showed that the medium contained less than 10 nM zinc. The medium was added with 15 g agar and sterilized at 121°C for 20 min.

At the same time, we constructed zinc deficiency SDA medium (SDA-Zn-2) by using TPEN (N,N,N′,N′-tetrakis(2-pyridylmethyl)ethylenediamine) (Cho et al., 2007; Thokala et al., 2019). TPEN (0.1 mM) was added into the liquid SDA medium (no agar). The contents of zinc, magnesium, copper, manganese, iron, potassium, and calcium were determined using atomic absorption spectroscopy to ensure zinc chelation (less than 10 nM), and other metal ions were added at recommended (Bio101) final concentrations. The medium was added with 15 g agar and sterilized at 121°C for 20 min.

Zinc sulfate was added into SDA-Zn-1 and SDA-Zn-2, and the final concentrations of zinc ions were 200 nM (Zn200), 400 nM (Zn400), 600 nM (Zn600), 800 nM (Zn800), and 1000 nM (Zn1000), respectively. Fungal conidia suspension (100 μl) was inoculated onto SDA (Norm), Zn200, Zn400, Zn600, Zn800, and Zn1000. Culture conditions were 28°C for 14 days.

The *Agrobacterium tumefaciens* strain EHA105 was maintained at 28°C on solid Yeast Extract Broth (YEB) supplemented with 50 μg/ml rifampicin (Sigma-Aldrich, USA) and 25 μg/ml chloramphenicol (Sigma-Aldrich, USA) (Yamada et al., 2009). *A. tumefaciens* EHA105 carrying *pDHt/ZafA::hph* was grown on YEB supplemented with 50 μg/ml rifampicin, 25 μg/ml chloramphenicol, and 50 μg/ml kanamycin (Sigma-Aldrich, USA). *A. tumefaciens*-induced medium (AIM), which was used for culturing *A. tumefaciens* carrying *pDHt/ZafA::hph* binary vectors, was supplemented with 50 μg/ml rifampicin, 25 μg/ml chloramphenicol, 50 μg/ml kanamycin, and 200 μM acetosyringone (Sigma-Aldrich, USA) (Bundock et al., 1995). The *M. canis ZafA* mutant strain was selected on solid SDA supplemented with 600 μg/ml hygromycin B (Sigma-Aldrich, USA) and 200 μg/ml cephalexin (Sigma-Aldrich, USA).

### RNA-Seq

The total RNAs from the Norm, Zn200, and Zn800 groups were extracted using the TRIzol^®^ reagent (Invitrogen, USA) following the manufacturer’s protocol, and DNase I (Takara, Japan) was used to remove genomic DNA. The integrity and size distributions of RNA were checked using the Agilent 2100 Bioanalyzer (Agilent, USA) with an RNA integrity number and GE ImageQuant 350 (GE Healthcare, USA). Poly(A) mRNA was enriched by Oligo (dT) beads (Qiagen, Germany). The enriched mRNA was fragmented and reverse transcribed into first-strand cDNAs by using random hexamers. The DNA polymerase I (Thermo Fisher Scientific, USA), RNase, dNTPs, and buffer were used to synthesize the second-strand cDNA. The Qiagen Quick PCR extraction kit (Qiagen, Germany) was used to purify cDNA fragments, which were ligated to Illumina sequencing adapters. Ligation products were selected on the basis of size by using agarose gel electrophoresis and amplified using PCR. The library passed quality inspection by using the Agilent 2100 Bioanalyzer (Agilent, USA) and the ABI StepOnePlus Real-Time PCR System (Applied Biosystems, USA). Sequencing was conducted on the Illumina HiSeq™ 2000 by the SAGENE Biotechnology Co., Ltd. (Guangzhou, China). After sequencing, raw reads were saved as FASTQ format. High-quality clean reads were generated from the assembly library by filtering with the following rules: reads containing adapters, >5% unknown nucleotides (N), and >50% low quality (q-value ≤ 10) bases were removed. HISAT2 (Daehwan et al., 2015) was performed to align high-quality reads with the *M. canis* genome (gcf_000151145.1_asm15114v1). The HTseq intersection nonempty method (Simon et al., 2015) was adopted to further locate the reads aligning with the *M. canis* genome and the exon regions of genes. Gene expression was calculated through the fragments per kilo bases per million (FPKM) method (Roberts, 2011), by using the following formula: RPKM = (1,000,000 C)/(N × L/1 000). RPKM is the expression of gene A, C is the number of reads that are uniquely mapped to gene A, N is the total number of reads that are uniquely mapped to all genes, and L is the base number for gene A. The correlation of gene expression levels among samples is an important index to test experiment reliability and reasonable sample selection. The Pearson correlation coefficient (corrplot package, https://cran.r-project.org/web/packages/corrplot/index.html) reflects the similarity degree of expression patterns among samples. A correlation coefficient ≥ 0.8 indicated highly relevant correlation, 0.5 ≤ correlation coefficient < 0.8 indicated moderate correlation, and 0.3 ≤ correlation coefficient < 0.5 indicated low correlation. A correlation coefficient < 0.3 indicated that the correlation between the two variables was extremely weak and could be regarded as irrelevant.

The differential expression analysis software we used was DESeq2 (Love et al., 2014). The screening conditions for DEGs were fold change > 2 with q-value or false discovery rate (FDR) < 0.01. Next, the FPKM values of DEGs were considered as expression levels, and the R toolkit pheatmap software was used for hierarchical clustering analysis. We used the GOseq (Young et al., 2010) and Kyoto Encyclopedia of Genes and Genomes (KEGG) public database for the GO and pathway enrichment analyses. Hypergeometric testing was performed. The calculated p-value was subjected to FDR correction, and FDR ≤ 0.01 was set as threshold.

Twelve DEGs were chosen for validation purposes. The wild-type *M. canis* was used as control group, and β-actin was used as reference gene. All relevant primers are shown in **Supplementary Material 15**. Reactions were conducted in accordance with the Maxima SYBR Green/ROX qPCR Master Mix (Thermo Scientific, USA) manual, and RT-qPCR was performed using the StepOnePlus™ Real-Time PCR System (Applied Biosystems, USA). A total of three biological and three technical replicates were used to determine Ct values. The expression levels of the tested genes were determined from Ct values, which were calculated using the 2^-△△Ct^ method (Livak and Schmittgen, 2001).

### Constructing Transformation Vectors

The *pDHt/ZafA::hph* binary vector, which was used for insertional mutagenesis, was constructed by fusing the hygromycin B resistance gene (*hph*) and the left and right flanking sequences of the *ZafA* gene from *M. canis*. They were inserted simultaneously into the *pDHt/SK* plasmid previously digested with *Xho*I/*Hin*dIII by using the In-Fusion HD Cloning Kit (Takara, Japan) in strict accordance with the instruction manual (Sleight et al., 2010). The left and right flanking fragments (*ZafA*-I nucleotide position: -886 to 71; *ZafA*-II nucleotide position: 1818 to 2638) of *ZafA* and *hph* genes (1931 bp) of *pAN7-1* were PCR-amplified using *ZafAI-F*/*ZafAI-R*, *ZafAII-F*/*ZafAII-R*, and *HphF*/*HphR* primer pairs, respectively (primer sequences shown in **Supplementary Material 15**). All fragments that were amplified or inserted into the plasmid were verified by DNA sequencing.

### *Agrobacterium Tumefaciens*-Mediated Transformation

*A. tumefaciens* EHA105 carrying *pDHt/ZafA::hph* was grown on YEB supplemented with rifampicin (50 μg/ml), chloramphenicol (25 μg/ml), and kanamycin (50 μg/ml), at 28°C for 48 h; collected in 10 ml sterile 0.9% sodium chloride solution; collected in 10 ml liquid YEB (50 μg/ml kanamycin, 50 μg/ml rifamycin, and 25 μg/ml chloramphenicol); and cultured at 28°C for 48 h. After centrifugation (3,500 rpm), *A. tumefaciens* was suspended in liquid AIM to obtain an optical density of 0.7 at 660 nm. After adding 200 μM acetosyringone, *A. tumefaciens* suspensions were incubated with shaking (150 rpm) at 28°C for 6 h.

The wild-type *M. canis* conidia concentration was adjusted to 1 × 10^7^ CFU/ml. A mixture of 100 μl *A. tumefaciens* and 100 μl *M. canis* conidia suspensions was spread onto sterilized nylon membranes, placed on solid AIM, and incubated at 28°C in the dark for 60 h. Nylon membranes were transferred onto the solid SDA plate supplemented with 600 μg/ml hygromycin B and 50 μg/ml cephalexin and incubated at 28°C for 3 days (Mora-Lugo et al., 2014; Zhang et al., 2014). Next, fungal colonies were produced on nylon membranes, and 10 samples were randomly chosen from one solid SDA plate and cultivated on solid SDA supplemented with 600 μg/ml hygromycin B at 28°C for 14 days. The *M. canis ZafA* mutant was labeled ZafA-hph.

ZafA-hph was subjected to PCR and Southern blotting to confirm the successful disruption of the *ZafA* gene. Total DNAs from the wild-type *M. canis* and ZafA-hph strains were extracted from growing mycelia by using sterile acid-washed glass beads (Sigma) (Yamada et al., 2002). The inserted *hph* was detected using *hph-F*/*hph-R* primers (845 bp, primer sequences shown in **Supplementary Material 15**). Specific gene disruption was confirmed using *ZafAq-F*/*ZafAq-R* primers (804 bp, primer sequences shown in **Supplementary Material 15**). Southern blotting was used to determine whether the mutant strain showed successful homologous recombination. The hybridization probe used to detect the *ZafA* fragment should be replaced by *hph* if the mutant was successful in ZafA-hph. Probes were labeled with digoxigenin-dUTP by using the DIGD-110 Labeling Kit (Roche, Switzerland). The probe length was 521 bp. The primer sequence is shown in **Supplementary Material 15**. Total DNA was digested using *XhoI* and *EcoRV* and target fragments were obtained. Digested samples were electrophoretically separated on 1% (w/v) agarose gels and transferred onto Hybond N+ membranes (Pharmacia, USA). Hybridization and signal detection were conducted in accordance with the manufacturer’s instructions.

### Determination of Growth Ability

Wild-type *M. canis* and ZafA-hph strains were separately inoculated into liquid/solid SDA medium and liquid/solid SDA-Zn-2 with 200, 400, 600, 800, and 1000 nM zinc sulfate at 28°C for 14 days. The growth of each strain was then observed. The liquid medium was centrifuged, and the supernatant was discarded and weighed to evaluate the growth capacity of wild-type *M. canis* and ZafA-hph strains at different zinc concentrations.

### *In Vitro* Biodegradation of Hair

Healthy human hair, dog hair, cat hair, rabbit hair, and fox hair samples were cut into 1-cm pieces, sterilized, and used as substrates for biodegradation assays. Conidia suspensions (200 μl, 10^8^ CFU/ml) of wild-type *M. canis* and ZafA-hph strains were added into 10 ml mineral medium to assess fungal biodegradation in the five hair types (100 mg) at 28°C for 28 days (Al-Janabi et al., 2016). The degree of hair degradation was recorded.

### Determination of Zinc Absorption Capacity

The wild-type *M. canis* and ZafA-hph strains were maintained at 28°C on SDA for 14 days. Sterile sodium chloride solution (0.9%) was used to wash off the fungus for fungal liquid collection and concentration adjustment. The 100-μl fungal liquid was inoculated onto sterilized nylon membranes (Sigma, USA) and placed on solid SDA at 28°C for 14 days. Next, 0.2 g fungus was scraped from the nylon membrane surface, and the zinc concentration in the fungus was determined using inductively coupled plasma mass spectrometry (ICP-MS).

ZafA-hph and wild-type *M. canis* strains were cultured on SDA-Zn-2 with 200 nM zinc sulfate at 28°C for 14 days. The wild-type *M. canis* cultured in normal SDA was used as control, and β-actin was used as the reference gene. The expression levels of MCYG_04486 (zinc transporter, zupT) and MCYG_02504 (membrane zinc transporter, ZTR) were examined in ZafA-hph and wild-type *M. canis* strains.

At the same time, ZafA-hph and wild-type *M. canis* strains were cultured on normal SDA, and 20 DEGs with significantly increased expression in *M. canis* under low zinc concentration culture were selected for determination in ZafA-hph. The wild-type *M. canis* was used as control, and β-actin was used as reference gene.

### Animal Inoculation Test

All adult New Zealand rabbits purchased from Northwest Agriculture and Forest University Animal Laboratories (Xian, China) were reared, obtained, and housed in accordance with our institute’s laboratory animal requirements. All procedures and the study design were conducted in accordance with the Guide for the Care and Use of Laboratory Animals (Ministry of Science and Technology of China, 2006) and approved by the Animal Ethical and Welfare Committee of Northwest Agriculture and Forest University (Approval No: 2020168).

Fifteen rabbits (8 males, 7 females, age = 1–1.5 years, weight = 2–3 kg) were selected for inoculation tests. Rabbits were randomly divided into groups for infection with the wild-type *M. canis* strain (n = 6), ZafA-hph (n = 6), or negative controls (n = 3). An area on each animal’s abdomen was shaved 1 day prior to inoculation. Following routine sterilization procedures, 200-μl (1 × 10^8^ CFU/ml) conidia suspensions of the wild-type *M. canis* and ZafA-hph strains were inoculated separately into the skin within the shaved abdominal area, and sterile saline was the inoculum for control animals. All rabbits were housed in separate cages to avoid cross-contamination. After 14 days, three rabbits were randomly selected from each test group, and one animal was randomly selected from the control group. Skin biopsies with diameter of 8.5 mm were obtained surgically. Skin infections were evaluated histologically.

### Statistical Analyses

Assays were repeated thrice. The one-way analysis of variance was used for statistical comparisons among different groups. Tests were performed using the IBM SPSS Statistics 24 software (SPSS Inc., Chicago, IL, USA). p < 0.05 was considered statistically significant.

## Results

### Effect of Zinc Deficiency on the Growth of *M. canis*

**Figure 1:**
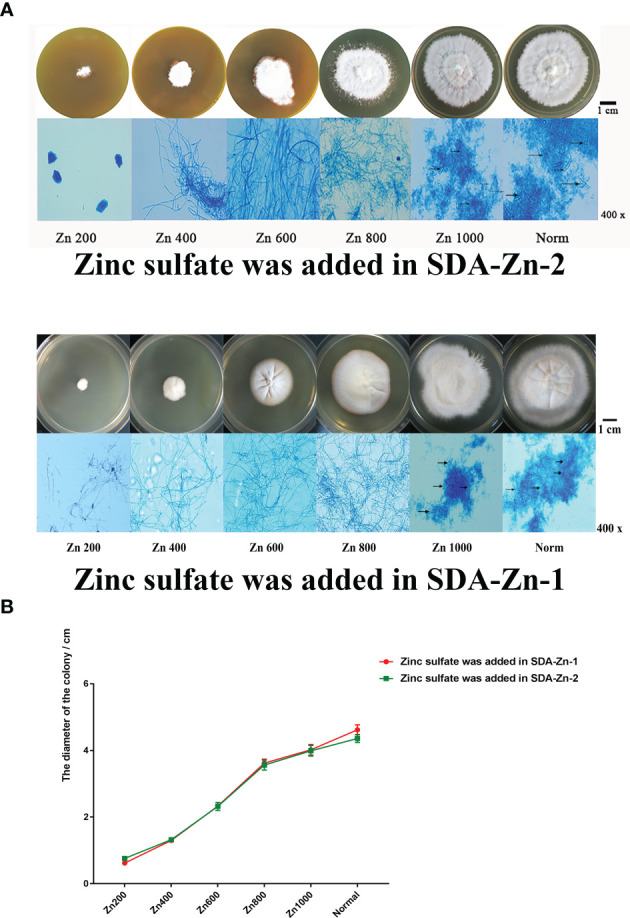
Growth situation of *M. canis* in normal SDA and zinc deficiency medium containing different zinc concentrations **(A)**. TPEN and Chelex-100 showed similar effects during zinc chelation according to the growth of *M. canis*. The diameters of colonies increased with increasing zinc concentrations for Zn200, Zn400, Zn600, Zn800, and Zn1000. No significant difference in colony morphology was observed between Zn1000 and Norm groups **(B)**. After lactate gossypol blue staining, no obvious conidium in Zn200, Zn400, Zn600, and Zn800 was seen upon microscopy, Zn1000 and Norm mycelia grew equally well, and macroconidia appeared. Black arrows indicate the macroconidia.

We constructed two kinds of zinc deficiency SDA (SDA-Zn-1 and SDA-Zn-2) by using Chelex-100 and TPEN. Zinc sulfate was added into SDA-Zn-1 and SDA-Zn-2, and the final concentrations of zinc ions were 200 nM (Zn200), 400 nM (Zn400), 600 nM (Zn600), 800 nM (Zn800), and 1000 nM (Zn1000), respectively*. M. canis* displayed differential growth in normal SDA medium and SDA-Zn-1/SDA-Zn-2 containing different zinc concentrations (**Figure 1A**). Macroscopically, white colonies were seen to radiate from the middle to the periphery of the culture, and their diameters increased with increasing zinc concentrations for Zn200, Zn400, Zn600, Zn800, and Zn1000 in two kinds of zinc deficiency SDA (**Figures 1A, B** **)**. No significant difference in colony morphology was observed between Zn1000 and Norm groups. In two kinds of zinc deficiency SDA, after lactate gossypol blue staining, no evident conidium in Zn200, Zn400, Zn600, and Zn800 groups was seen upon microscopy. Zn1000 and Norm mycelia grew equally well, and macroconidia appeared (**Figure 1A**). Results collectively indicated that when *M. canis* grew under zinc-deficient conditions, growth was adversely affected, showing that zinc was important for *M. canis*. At the same time, results showed that SDA-Zn-1 and SDA-Zn-2 could be successfully used to prepare Zn-deficient SDA and presented the same effect. In accordance with the growth of *M. canis*, we selected Zn200 (zinc sulfate was added into SDA-Zn-2), Zn800 (zinc sulfate was added in SDA-Zn-2), and Norm groups for transcriptome sequencing and investigated the effect of zinc deficiency on the gene expression and metabolism of *M. canis*.

### Sequencing Quality Control and Alignment Analysis

The concentration, purity, and integrity of RNA were detected (**Supplementary Material 1**). Sequence data were deposited in the NCBI Short Read Archive database (BioProject accession number: PRJNA563545; BioSample accession number: SAMN12676499; SRA accession numbers: SRR10064541 (Zn200-1), SRR10064540 (Zn200-1), SRR10064539 (Zn200-3), SRR10064538 (Zn800-1), SRR10064537 (Zn800-2), SRR10064536 (Zn800-3), SRR10064535 (Norm-1), SRR10064534 (Norm-2), and SRR10064533 (Norm-3). The sequencing quality is shown in **Supplementary Material 2**. The proportion of reads that aligned with the *M. canis* reference genome is shown in **Supplementary Material 2**. The proportion was 9.27% for NORM_1, indicating that the sample was contaminated and therefore eliminated from the subsequent analysis. The alignment proportions of NORM_2, NORM_3, Zn200_1, Zn200_2, Zn200_3, Zn800_1, Zn800_2, and Zn800_3 were 93.49%, 92.85%, 96.17%, 96.02%, 95.37%, 96.44%, 96.56%, and 96.04%, respectively, making them suitable for subsequent data analysis.

### Quantitative Expression and Differential Gene Expression Analyses

**Figure 2:**
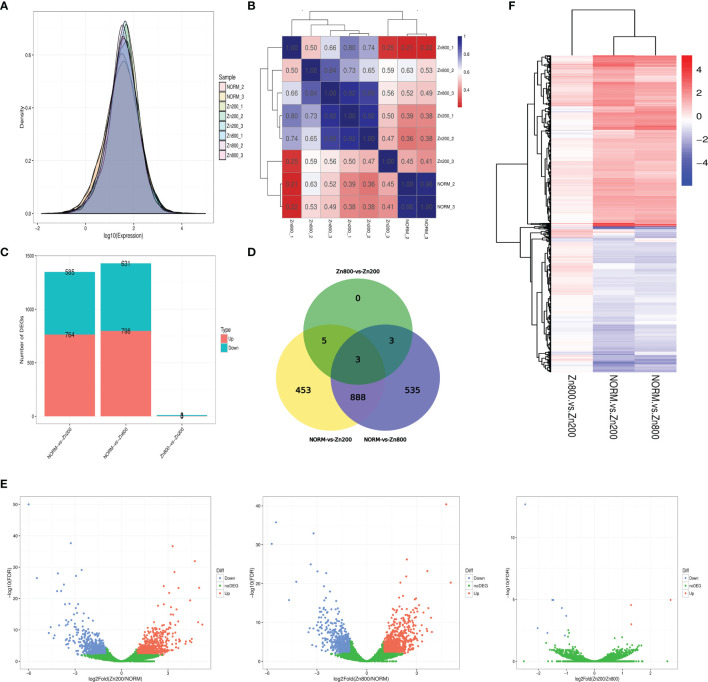
RAN-seq results. **(A)** Gene expression density distributions of NORM_2, NORM_3, Zn200_1, Zn200_2, Zn200_3, Zn800_1, Zn800_2, and Zn800_3. The X-axis represents the sample name; the Y-axis represents the gene expression levels (logarithm for base 10). **(B)** Pearson correlation coefficient between each sample. The X-axis represents the sample name; the Y-axis represents the sample name. Blue and red colors indicate high and low correlations, respectively, between two samples. **(C)** Statistics on the number of DEGs between samples. The X-axis represents the comparison between samples, and the Y-axis represents the number of DEGs. Red and blue colors represent upregulated and downregulated genes, respectively, in **(C)**. **(D)** Venn diagram of DEGs. Seven hundred sixty-four genes were upregulated and 585 were downregulated in NORM-*vs*-Zn200. In NORM-*vs*-Zn800, 798 genes were upregulated and 631 were downregulated. There were three upregulated genes and eight downregulated genes observed in Zn200-*vs*-Zn800. **(E)** Volcano map of the DEGs between the different groups. The X-axis represents fold change, and the Y-axis represents -log10 (q-value). Each dot represents a gene, the blue (downregulated) and red (upregulated) dots represent DEGs, and the green dots indicate that the genes are not significantly different. **(F)** Cluster analysis on the DEGs. Each column represents an experimental condition, and each row represents a gene. Color indicates the expression amount or fold change. Red and blue colors indicate high and low expression amounts or fold changes, respectively.

In the RNA-seq analysis, we used the FPKM method to calculate each gene expression quantity (**Supplementary Material 3**). The gene expression density distributions of NORM_2, NORM_3, Zn200_1, Zn200_2, Zn200_3, Zn800_1, Zn800_2, and Zn800_3 are shown in **Figure 2A**, and the Pearson correlation coefficient between each sample is shown in **Figure 2B**.

According to the statistics for the number of DEGs, 764 genes were upregulated and 585 were downregulated in NORM-*vs*-Zn200. Three DEGs, MCYG_04486 (zinc transporter, zupT), MCYG_06235 (zinc-responsiveness transcriptional activator, ZafA), and MCYG_02504 (membrane zinc transporter, ZTR), which were possibly related to zinc ion absorption in *M. canis*, were significantly upregulated. In NORM-*vs*-Zn800, 798 genes were upregulated and 631 genes were downregulated; MCYG_04486 and MCYG_06235 were also significantly upregulated. A total of three upregulated genes and eight downregulated genes were observed in Zn200-*vs*-Zn800 (**Supplementary Material 4** and **Figures 2C, D** **)**. A volcano map of DEGs among different groups is shown in **Figure 2E**. The DEG analysis showed that zinc deficiency significantly affected gene expression in *M. canis*. We also performed a cluster analysis on DEGs and experimental conditions by using the R toolkit pheatmap software (**Figure 2F** and **Supplementary Material 5**).

### GO and KEGG Pathway Analyses of DEGs

From the GO enrichment analysis on all DEGs, we selected DEGs with the 30 most significant enrichment GO terms on the basis of p-values (**Supplementary Material 6**). On the basis of the p-values of the upregulated and downregulated DEGs corresponding to GO terms, we selected 10 most significantly enriched GO terms from three GO aspects, from which the number of upregulated and downregulated DEGs corresponding to each GO term was counted (**Supplementary Material 7**). In the GO enrichment of DEGs from NORM-*vs*-Zn200, the terms with the largest numbers of enriched genes were the nucleus, the cytosol, integral membrane component, cytoplasm, plasma membrane, nucleotide binding, and zinc ion binding, among others. The terms with the largest numbers of enriched genes in DEGs for NORM-*vs*-Zn800, were the nucleus, cytosol, integral membrane component, cytoplasm, zinc ion binding, membrane, nucleotide binding, and plasma membrane, among others (**Supplementary Material 8**). Many DEGs were enriched in GO terms related to cellular structure, and DEGs enriched in zinc ion binding might play an important role in zinc absorption by *M. canis*.

We also found that many DEGs in the GO functional enrichment analysis were enriched in GO terms related to growth and oxidative stress in *M. canis*. These DEGs included hyphal growth, filamentous growth of a population of unicellular organisms in response to starvation, filamentous growth of a population of unicellular organisms, sporocarp development associated with sexual reproduction, nucleotide binding, and cellular response to oxidative stress, among others (**Supplementary Material 8**). The GO function analysis indicated that zinc deficiency significantly affected gene expression and growth in *M. canis*.

Genes usually play roles in biological functions by interacting with each other, and the KEGG pathway analysis can enhance our understanding of the biological functions of genes. In the KEGG pathway analysis of NORM-*vs*-Zn200, 774 DEGs were annotated in 110 pathways; pathways with the highest numbers of annotated DEGs were metabolic pathways, biosynthesis of secondary metabolites, biosynthesis of antibiotics, and purine metabolism, among others. In NORM-*vs*-Zn800, 822 DEGs were annotated in 112 pathways; pathways with the highest numbers of annotated DEGs included biosynthesis of secondary metabolites, biosynthesis of antibiotics, yeast cell cycle, and yeast meiosis, among others. In the KEGG pathway analysis of Zn800-*vs*-Zn200, two DEGs were annotated in six pathways (**Supplementary Material 9**). On the basis of p-values, we selected the most enriched 30 pathways from the pathway analysis results and counted the number of upregulated and downregulated DEGs for each term (**Supplementary Material 10**). Many DEGs were enriched in KEGG pathways related to the cell growth cycle, biosynthesis of antibiotics, and nutrition metabolism. Results indicated that zinc deficiency significantly affected the growth and virulence of *M. canis*, resulting in low resistance of *M. canis* to the environment.

### Validation of RT-qPCR

The 12 selected DEGs were verified by RT-qPCR. MCYG_04486, MCYG_06235, and MCYG_02504, which might be related to zinc ion absorption, were significantly upregulated compared with those in the Norm group. The other nine DEGs, including five upregulated DEGs (MCYG_00110, MCYG_04543, MCYG_07841, MCYG_01475, MCYG_05608) and four downregulated DEGs (MCYG_02286, MCYG_07837, MCYG_04785, MCYG_08408), also showed significant differences. These results matched those obtained from our RNA-seq analysis (**Supplementary Material 11**).

### Confirmation of the *M. canis ZafA* Gene Mutation

The *ZafA* gene was significantly upregulated in Zn200 and Zn800 groups and shared homology with *Zap1*. Thus, the *ZafA* gene might be the main transcription factor regulating *M. canis* zinc homeostasis. However, the function of *ZafA* gene was unclear and should be verified. The *hph* gene and the upstream and downstream flanking sequences of the *ZafA* gene were simultaneously inserted into the *Xho*I*/Hin*dIII digested *pDHt/SK* plasmid *via* the In-Fusion HD cloning kit (**Supplementary Material 12**). The binary *pDHt/ZafA::hph* carrier obtained was transformed into *A. tumefaciens* EHA105, and its successful transformation was validated (**Supplementary Material 13**).

A total of 100 colonies were selected and inoculated onto SDA containing hygromycin B. PCR and Southern blotting were performed to confirm that *pDHt/ZafA::hph* underwent homologous recombination in the recipient wild-type *M. canis* strain. PCR indicated that *hph* was inserted into ZafA-hph as determined by its successful amplification in 90% of strains. The *hph*-positive strains were PCR-verified using *ZafAq-F/ZafAq-R*-specific primers (**Supplementary Material 15**) with the *ZafA* gene deletion sequence, and 2% of these strains were negative. Southern blotting, which was performed to further confirm that homologous recombination occurred, showed that *ZafA* was disrupted in ZafA-hph. These results indicated that the *ZafA* gene was successfully mutated in ZafA-hph (**Supplementary Material 13**).

### Growth Abilities of the ZafA-hph Strain

**Figure 3:**
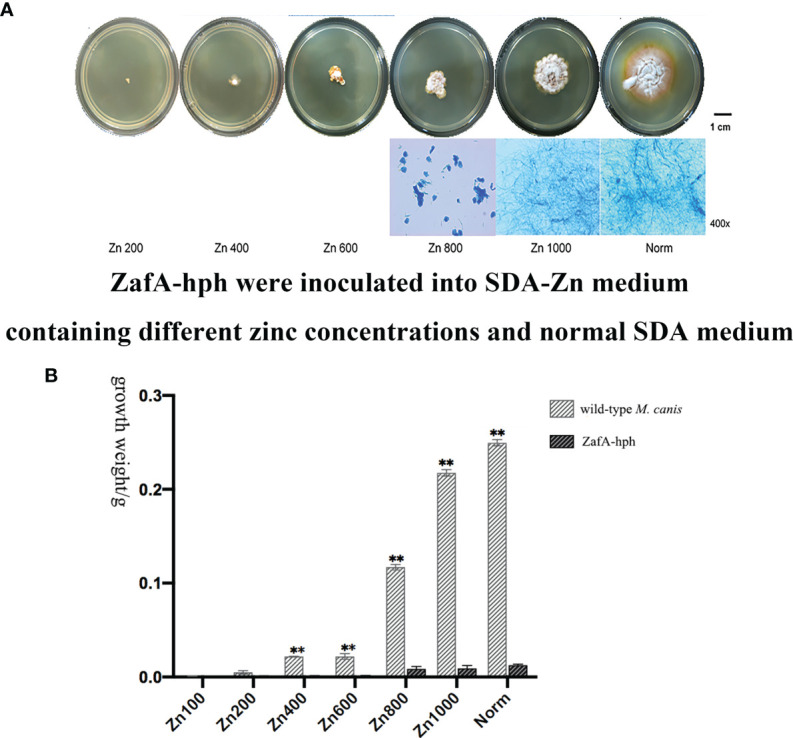
Growth abilities of the ZafA-hph strain. **(A)** The ZafA-hph inoculated into SDA-Zn-2 containing different zinc concentrations and normal SDA at 28°C for 14 days. In SDA-Zn and normal SDA, the growth, colony diameter, and reproduction ability of ZafA-hph were significantly lower than those of the wild-type *M. canis* strain, and no macroconidia was formed in ZafA-hph. **(B)** Extremely significantly low growth performance and weight of ZafA-hph than those of the wild-type *M. canis* strain, ^∗∗^ *p < 0.01*.

ZafA-hph was inoculated onto SDA-Zn-2 containing different zinc concentrations and normal SDA at 28°C for 14 days. In the Norm and Zn1000 groups, the colonial morphology of ZafA-hph showed a yellow border and differed from that of the wild-type *M. canis* strain. The diameters of ZafA-hph colonies were significantly smaller than those of the wild-type *M. canis* strain. In Zn200, Zn400, Zn600, and Zn800 groups, the growth, colony diameter, and reproduction ability of ZafA-hph were significantly lower than those of the wild-type *M. canis* strain. No evident filamentous structure was observed, but clumpy aggregation and growth were observed (**Figure 3A**). Microscopically, the number of hyphae in ZafA-hph was significantly lower than that of the wild-type *M. canis* strain grown under different culture conditions, and no conidia was formed in ZafA-hph. In Zn800, no hypha or spore formation occurred in ZafA-hph, and only a flake fungus block was seen, which could not be separated into hyphae (**Figure 3A**).

In SDA, ZafA-hph and wild-type *M. canis* strains differed significantly in growth performance and weight (**Figure 3B**). These results showed that the growth performance of ZafA-hph decreased significantly compared with that of the wild-type strain, which confirmed that the *ZafA* gene played an important role in the growth of *M. canis.*

### Zinc Absorption Capacities of the ZafA-hph Strain

**Figure 4:**
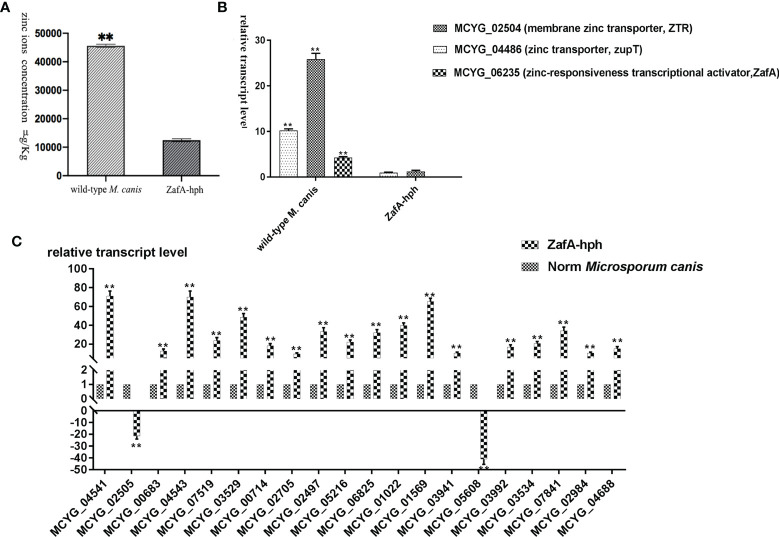
Zinc absorption capacity of ZafA-hph. **(A)** Significantly lower zinc ion concentration in ZafA-hph relative to the wild-type *M. canis* strain, ^∗∗^ *p < 0.01*. **(B)** Significantly reduced expression levels of MCYG_04486 and MCYG_02504 in ZafA-hph compared with those in the wild-type *M. canis* strain, ^∗∗^ *p < 0.01*. **(C)** Decreased MCYG_02505 and MCYG_05608 and 18 significantly increased DEGs. Results were basically consistent with those of sequencing results. The lack of zinc ion could lead to a significant increase in the expression of these genes, which also confirmed the decreased zinc ion concentration in ZafA-hph, ^∗∗^ *p < 0.01*.

The zinc ion concentrations of wild-type *M. canis* and ZafA-hph strains were determined using ICP-MS. Relative to that in the wild-type *M. canis* strain, the zinc ion concentration in ZafA-hph was significantly lower (**Figure 4A**). These results showed that the absence of *ZafA* could significantly negatively affect *M. canis* zinc absorption ability.

The expression levels of MCYG_04486 (zinc transporter, zupT) and MCYG_02504 (membrane zinc transporter, ZTR) in ZafA-hph were significantly reduced compared with those in the wild-type *M. canis* strain (**Figure 4B**).

We selected 20 DEGs with significantly increased expression in *M. canis* under low zinc concentration culture for determination in ZafA-hph (**Supplementary Material 14**, primer sequences shown in **Supplementary Material 15**). Among the 20 DEGs, only MCYG_02505 and MCYG_05608 were decreased, whereas the 18 other DEGs were significantly increased. These results were basically consistent with the sequencing results (**Figure 4C**). The lack of zinc ion could lead to a significant increase in the expression of these genes, which also confirmed the decrease of zinc ion concentration in ZafA-hph. MCYG_02505 and MCYG_05608 were decreased, which might be related to the expression of the *ZafA* gene. The expression levels of these two genes were decreased due to the knockout of the *ZafA* gene.

### *In Vitro* Biodegradation of Hair

**Figure 5:**
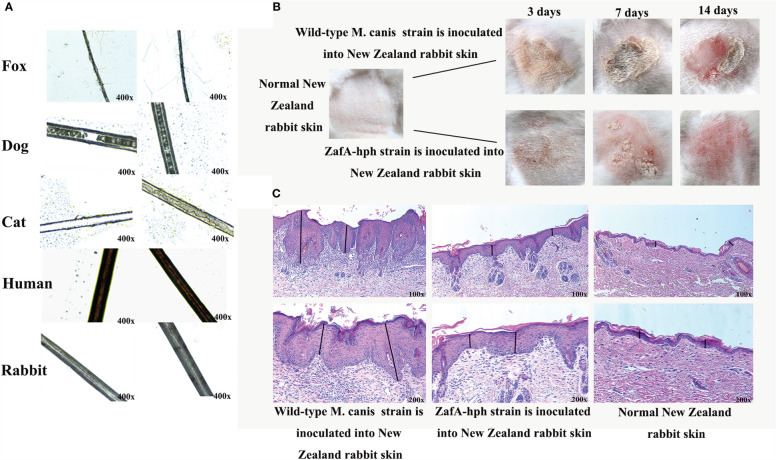
Virulence test of ZafA-hph. **(A)** No evident pathological change or decomposition in human or rabbit hair. Dog and cat hairs were highly susceptible to *M. canis*, and the decomposition was significantly reduced on dog and cat hairs in ZafA-hph. The fox hair was slightly susceptible to *M. canis*, and the hair appeared to be slightly damaged. No damage was apparent with the ZafA-hph infection. **(B)** Serious skin damage caused by wild-type *M. canis* in 3, 7, and 14 days and normal skin observed in ZafA-hph in 14 days. **(C)** Compared with normal rabbit skin, different degrees of thickening of the stratum corneum and stratum spinosum layers were observed, with initial inflammatory cell infiltration apparent after inoculation with wild-type *M. canis*. Rabbit skin showed slight lesions and slight thickening of the stratum corneum and spinous layer after inoculation with ZafA-hph but with no evident difference from normal skin. The black line represents stratum corneum and stratum spinosum layers.

Wild-type *M. canis* and ZafA-hph strains were cocultured with different hair types in mineral culture medium. No evident pathological change or decomposition was found in human or rabbit hair, suggesting low susceptibility to the *M. canis* test isolate. However, dog and cat hairs were sensitive to infection with the wild-type *M. canis* strain. Notably, hair was visibly damaged and perforated, but the degree of hair damage was significantly reduced without evident abnormality under the ZafA-hph infection. Fox hair was slightly sensitive to infection with wild-type *M. canis* strain. In this case, the hair appeared slightly damaged, but no damage was apparent with the ZafA-hph infection (**Figure 5A**). Overall, the infection with ZafA-hph significantly reduced the biodegradation of hair in dogs, cats, and foxes compared with wild-type *M. canis* strain infection, indicating that the *ZafA* gene played an important role in hair degradation from wild-type *M. canis*.

### Animal Skin Inoculation Test

The wild-type *M. canis* and ZafA-hph strains were inoculated into New Zealand rabbit skin to compare their pathogenic abilities. Wild-type *M. canis* could cause serious skin damage, but the skin was basically back to normal in ZafA-hph in 14 days (**Figure 5B**). Compared with normal rabbit, rabbits inoculated with wild-type *M. canis* had different degrees of thickening of the stratum corneum and stratum spinosum layers with initial inflammatory cell infiltration. Rabbit skin showed slight lesions and slight thickening of the stratum corneum and spinous layer after inoculation with ZafA-hph, but had no evident difference from normal skin (**Figure 5C**). This result indicated that the *ZafA* gene played an important role in the pathogenicity of *M. canis*.

## Discussion

*M. canis*, one of the most common dermatophytes, can infect the skin, hair, and nails and cause superficial mycosis (Abastabar et al., 2019). In-depth studies on this fungus are warranted, and RNA-seq provides a new way to study pathogenic fungi. This tool has been widely used in the study of dermatophytes, such as *Trichophyton mentagrophyte* and *T. rubrum* (Mendes et al., 2018; Petrucelli et al., 2018; Xiao et al., 2018). In 2017, RNA-seq was conducted on *T. mentagrophyte* cultured under different zinc ion concentrations, and genes related to zinc absorption are identified. The importance of zinc to the growth and virulence of *T. mentagrophyte* is confirmed (Zhang et al., 2017).

Currently, the effects of zinc deficiency on the growth, virulence, gene expression, zinc absorption, and biological metabolism of *M. canis* remain unknown. Therefore, in the present study, we constructed two kinds of zinc deficiency SDA (other metal ions were added at recommended (Bio101) final concentrations, and the concentration of zinc ions was below 10 nM). We cultured *M. canis* under different zinc ion concentrations, and under increasing zinc ion concentrations in a zinc-deficient medium the growth of *M. canis* tended to be normal. When the concentration of zinc ions added to the zinc-deficient medium reached 1000 nM, the growth characteristics of *M. canis* were not significantly different from those of *M. canis* in normal medium. However, the differences in the growth characteristics observed in Zn200, Zn400, Zn600, and Zn800 groups were caused by zinc ions. Zinc ions are essential for good growth in *M. canis*, indicating that a mechanism exists for zinc absorption and transport in *M. canis*. In addition, we used Chelex-100 and TPEN to prepare zinc deficiency medium, and results showed that Chelex-100 and TPEN could be successfully used for the preparation of zinc deficiency medium and had the same effect. The dose used in this study did not cause cell toxicity. This finding was similar to the conclusions of other researchers (Lyons et al., 2000; Cho et al., 2007; Hamon et al., 2014; Thokala et al., 2019; Yu and Yu, 2019). Finally, in accordance with the observed growth differences of *M. canis* in different zinc concentrations, Zn200, Zn800 and Norm groups were selected for transcriptome sequencing analysis.

Zinc is extremely important for the growth of fungi, and zinc chelation can restrict the growth of fungi in any medium (Lulloff et al., 2004). Yeast in *S. cerevisiae* responds to the growth stress caused by zinc deficiency through a variety of different strategies (Staats et al., 2013). The transcriptome and functional analyses of *S. cerevisiae* show that low zinc conditions can lead to changes in lipid synthesis, amino acid metabolism, sulfate metabolism, and oxidative stress tolerance (Iwanyshyn et al., 2004; Carman and Han, 2007). Growth and gene expression in *A. fumigatus*, *C. gattii*, and *C. albicans* can also be negatively affected by the absence of zinc ions in the medium (Moreno et al., 2007; Kim et al., 2008; Schneider Rde et al., 2012; Jung, 2015). Similar results were also found in the present study, and according to our DEG analysis, many genes showed significant upregulation and downregulation respectively in *M. canis* with zinc deficiency. In the GO functional enrichment analysis, we found that many DEGs were enriched in GO terms related to cellular structure, growth, and oxidative stress in *M. canis*. In the KEGG pathway analysis, many DEGs were enriched in KEGG pathways related to the cell growth cycle, biosynthesis of antibiotics, and nutrition metabolism. This result indicated that zinc deficiency could affect the gene expression and various metabolic processes of *M. canis* and hinder its growth and reproduction.

The mechanism underlying zinc absorption transport, which is first determined in *S. cerevisiae*, involves *Zap1* gene regulation (Eide, 1998). The compositions and functions of the zinc response activity factors (Zap1 homologs) have been determined for various fungi, such as the ZafA protein in *A. fumigatus* (Moreno et al., 2007). In the present study, *ZafA* was significantly upregulated in Zn200 and Zn800 and shared homology with *Zap1*. Thus, *ZafA* might be the main transcription factor regulating *M. canis* zinc homeostasis. However, its specific function remains elusive, and its influence on the growth and virulence of *M. canis* is unknown. Therefore, we constructed a *ZafA* gene knockout strain of *M. canis* to verify its specific function in *M. canis* and confirm its importance to this fungus. The expression levels of MCYG_04486 (zinc transporter zupT) and MCYG_02504 (membrane zinc transporter, ZTR) were significantly reduced in ZafA-hph and could be *ZafA*’s target gene. Thus, more research is needed. DEGs enriched in zinc ion binding might also play an important role in zinc absorption by *M. canis*, but more research is needed.

ATMT is a common gene manipulation method for filamentous fungi. In 2009, Yamada first completed the genetic transformation of *T. mentagrophyte via* ATMT (Yamada et al., 2009). Next, Zhang and Shi successfully transformed the *Mep1-5* and *Sub6* genes from *T. mentagrophyte via* the *A. tumefaciens* EHA105 strain (Zhang et al., 2014; Shi et al., 2016). In our study, the *A. tumefaciens* EHA105 strain was selected to complete the genetic transformation of the *M. canis ZafA* gene. A stable *ZafA* gene knockout strain was obtained, and the homologous recombination efficiency was only 2%, which was lower than that obtained for other fungi (Zeilinger, 2004). This result might be related to the difference between strains and specific gene locus, and the target gene locus might affect the homologous recombination rate (Bird and Bradshaw, 1997). As this is the first time that ATMT has been used in *M. canis*, the present study provides a reference for selecting genetic manipulation tools for this fungus, but more studies are needed to improve the homologous recombination efficiency.

In the present study, the growth performance, hair degradation capacity, and pathogenicity of the *ZafA* gene knockout strain of *M. canis* decreased significantly compared with the wild-type strain, which showed that this gene played an important role in growth, reproduction, and pathogenicity of *M. canis*. Relative to that in the wild-type *M. canis* strain, the zinc ion concentration in ZafA-hph was significantly lower, which showed that the absence of *ZafA* could significantly negatively affect *M. canis* zinc absorption ability. We selected 20 DEGs with significantly increased expression in *M. canis* under low zinc concentration culture for determination in ZafA-hph. This result was consistent with sequencing results. The lack of zinc ion could lead to a significant increase in the expression of these genes, which also confirmed the decrease of zinc ion concentration in ZafA-hph. MCYG_02505 and MCYG_05608 were decreased, which might be related to the expression of the *ZafA* gene. The expression of these two genes was decreased due to the knockout of the *ZafA* gene. Thus, more research is needed. The *ZafA* gene can be used as a new drug target. Cutting off the zinc absorption pathway can be used as a way to prevent and control infection in *M. canis*. We can try to find one or more drugs that can block the synthesis of ZafA protein to inhibit the growth of *M. canis* and reduce its virulence. Moreover, the ZafA protein has no homologous protein in the human body and can be considered as immunogen for the development of vaccine.

## Conclusion

The growth of *M. canis* is severely inhibited, and many genes show significant upregulation and downregulation in *M. canis* with zinc deficiency. Zinc deficiency can negatively affect gene expression and biological metabolic pathway in *M. canis*. The *ZafA* gene may be the main transcription factor regulating *M. canis* zinc homeostasis. ATMT can be successfully used to transform the *ZafA* gene from *M. canis*, and a stable *ZafA* gene knockout is obtained. The growth performance, hair degradation ability, virulence, zinc absorption capacity, and expression of zinc transporter genes of ZafA-hph are lower than those of the wild-type *M. canis*. The *ZafA* gene can be used as a new drug target.

## Data Availability Statement

The datasets presented in this study can be found in online repositories. The names of the repository/repositories and accession number(s) can be found in the article/**Supplementary Material**.

## Ethics Statement

All procedures and the study design were conducted in accordance with the Guide for the Care and Use of Laboratory Animals (Ministry of Science and Technology of China, 2006) and were approved by the Animal Ethical and Welfare Committee of Northwest Agriculture and Forest University (Approval No. 2020168).

## Author Contributions

XZ, PD, and YL conceived the experiments and performed the experiment. PD and XZ carried out the data analysis and wrote the paper. YL, JH, PG, HX, and YZ helped to carry out the data analysis and participated in the drafted manuscript. All authors contributed to the article and approved the submitted version.

## Funding

This work was supported by the Youth Science Fund Project of the National Natural Science Foundation of China (project number: 31402262) in the design of the study and collection, in the analysis of data, and in writing the manuscript, and the Program of Shaanxi Province Science and Technology Innovation Team (project number: 2019TD-036) in the collection, analysis, and interpretation of data.

## Conflict of Interest

The authors declare that the research was conducted in the absence of any commercial or financial relationships that could be construed as a potential conflict of interest.

## Publisher’s Note

All claims expressed in this article are solely those of the authors and do not necessarily represent those of their affiliated organizations, or those of the publisher, the editors and the reviewers. Any product that may be evaluated in this article, or claim that may be made by its manufacturer, is not guaranteed or endorsed by the publisher.

## Supplementary Material

Supplementary Material 1 The concentration, purity and integrity of RNA.

Supplementary Material 2 The sequencing quality and the proportion of reads that aligned with the M. canis reference genome.

Supplementary Material 3 Each gene expression quantity.

Supplementary Material 4 The DEGs among different samples.

Supplementary Material 5 The cluster analysis

Supplementary Material 6 The DEGs with the 30 most significant enrichment GO terms, based on the P-value values.

Supplementary Material 7 The number of up- and down-regulated DEGs corresponding to each GO term.

Supplementary Material 8 The GO enrichment of DEGs.

Supplementary Material 9 The KEGG pathway analysis of DEGs.

Supplementary Material 10 The DEGs with the 30 most significant pathways, based on the P-value values.

Supplementary Material 11 The validation of RT-qPCR.

Supplementary Material 12 The construction of the transformation vectors.

Supplementary Material 13 The validation of *pDHt/ZafA::hph and ZafA-hph*.

Supplementary Material 14 20 DEGs with significantly increased expression in M. Canis under low zinc concentration culture.

Supplementary Material 15 The sequences of primers.

## References

[B1] Abastabar M. Jedi A. Guillot J. Ilkit M. Eidi S. Hedayati M. T.. (2019). *In Vitro* Activities of 15 Antifungal Drugs Against a Large Collection of Clinical Isolates of Microsporum Canis. Mycoses 62 (11), 1069–1078. doi: 10.1111/myc.12986 31408550

[B2] Achterman R. R. White T. C. (2012). A Foot in the Door for Dermatophyte Research. PloS Pathog. 8, e1002564. doi: 10.1371/journal.ppat.1002564 22479177 PMC3315479

[B3] Al-Janabi A. Ai-Tememi N. N. Ai-Shammari R. A. Ai-Assadi A. H. A. (2016). Suitability of Hair Type for Dermatophytes Perforation and Differential Diagnosis of T. Mentagrophytes From T. Verrucosum. Mycoses 59, 247–252. doi: 10.1111/myc.12458 26776657

[B4] Bird D. Bradshaw R. (1997). Gene Targeting is Locus Dependent in the Filamentous Fungus Aspergillus Nidulans. Mol. Gen. Genet. 255, 219–225. doi: 10.1007/s004380050492 9236780

[B5] Broshnissimov T. Benami R. Astman N. Malin A. Baruch Y. Galor I. (2018). An Outbreak of Microsporum Canis Infection at a Military Base Associated With Stray Cat Exposure and Person-to-Person Transmission. Mycoses 61 (7), 472–476. doi: 10.1111/myc.12771 29570867

[B6] Bundock P. den Dulk-Ras A. Beijersbergen A. Hooykaas P. J. (1995). Trans-Kingdom T-DNA Transfer From Agrobacterium Tumefaciens to Saccharomyces Cerevisiae. EMBO J. 14, 3206–3214. doi: 10.1002/j.1460-2075.1995.tb07323.x 7621833 PMC394382

[B7] Carman G. M. Han G. S. (2007). Regulation of Phospholipid Synthesis in Saccharomyces Cerevisiae by Zinc Depletion. Biochim. Biophys. Acta 1771, 322–330. doi: 10.1016/j.bbalip.2006.05.006 16807089 PMC1876696

[B8] Cho Y. E. Lomeda R. A. Ryu S. H. Lee J. H. Beattie J. H. Kwun I. S. (2007). Cellular Zn Depletion by Metal Ion Chelators (TPEN, DTPA and Chelex Resin) and Its Application to Osteoblastic MC3T3-E1 Cells. Nutr. Res. Pract. 1, 29–35. doi: 10.4162/nrp.2007.1.1.29 20535382 PMC2882573

[B9] Daehwan K. Ben L. Salzberg S. L. (2015). HISAT: A Fast Spliced Aligner With Low Memory Requirements. Nat. Methods 12, 357–360. doi: 10.1038/nmeth.3317 25751142 PMC4655817

[B10] Eide D. J. (1998). The Molecular Biology of Metal Ion Transport in Saccharomyces Cerevisiae. Annu. Rev. Nutr. 18, 441–469. doi: 10.1146/annurev.nutr.18.1.441 9706232

[B11] Hamon R. Homan C. C. Tran H. B. Mukaro V. R. Lester S. E. Roscioli E.. (2014). Zinc and Zinc Transporters in Macrophages and Their Roles in Efferocytosis in COPD. PloS One 9, e110056. doi: 10.1371/journal.pone.0110056 25350745 PMC4211649

[B12] Iwanyshyn W. M. Han G. S. Carman G. M. (2004). Regulation of Phospholipid Synthesis in Saccharomyces Cerevisiae by Zinc. J. Biol. Chem. 279, 21976–21983. doi: 10.1074/jbc.M402047200 15028711

[B13] Jung W. H. (2015). The Zinc Transport Systems and Their Regulation in Pathogenic Fungi. Mycobiology 43, 179–183. doi: 10.5941/MYCO.2015.43.3.179 26539032 PMC4630422

[B14] Kim M. J. Kil M. Jung J. H. Kim J. (2008). Roles of Zinc-Responsive Transcription Factor Csr1 in Filamentous Growth of the Pathogenic Yeast Candida Albicans. J. Microbiol. Biotechnol. 18, 242–247. 18309267

[B15] Livak K. J. Schmittgen T. D. (2001). Analysis of Relative Gene Expression Data Using Real-Time Quantitative PCR and the 2(-Delta Delta C(T)) Method. Methods (San Diego Calif.) 25, 402–408. doi: 10.1006/meth.2001.1262 11846609

[B16] Love M. I. Huber W. Anders S. (2014). Moderated Estimation of Fold Change and Dispersion for RNA-Seq Data With DESeq2. Genome Biol. 15, 550. doi: 10.1186/s13059-014-0550-8 25516281 PMC4302049

[B17] Lulloff S. J. Hahn B. L. Sohnle P. G. (2004). Fungal Susceptibility to Zinc Deprivation. J. Lab. Clin. Med. 144, 208–214. doi: 10.1016/j.lab.2004.07.007 15514589

[B18] Lyons T. J. Gasch A. P. Gaither L. A. Botstein D. Brown P. O. Eide D. J. (2000). Genome-Wide Characterization of the Zap1p Zinc-Responsive Regulon in Yeast. Proc. Natl. Acad. Sci. U. S. A. 97, 7957–7962. doi: 10.1073/pnas.97.14.7957 10884426 PMC16652

[B19] Mendes N. S. Bitencourt T. A. Sanches P. R. Silva-Rocha R. Martinez-Rossi N. M. Rossi A. (2018). Transcriptome-Wide Survey of Gene Expression Changes and Alternative Splicing in Trichophyton Rubrum in Response to Undecanoic Acid. Sci. Rep. 8, 2520. doi: 10.1038/s41598-018-20738-x 29410524 PMC5802734

[B20] Mora-Lugo R. Zimmermann J. Rizk A. M. Fernandez-Lahore M. (2014). Development of a Transformation System for Aspergillus Sojae Based on the Agrobacterium Tumefaciens-Mediated Approach. BMC Microbiol. 14, 247. doi: 10.1186/s12866-014-0247-x 25253558 PMC4186950

[B21] Moreno M. A. Ibrahim-Granet O. Vicentefranqueira R. Amich J. Ave P. Leal F.. (2007). The Regulation of Zinc Homeostasis by the ZafA Transcriptional Activator Is Essential for Aspergillus Fumigatus Virulence. Mol. Microbiol. 64, 1182–1197. doi: 10.1111/j.1365-2958.2007.05726.x 17542914

[B22] Nakamura Y. Watanabe S. Hasegawa A. (1999). Dermatomycosis in Human and Animals. Nippon Ishinkin Gakkai Zasshi 40, 9–14. doi: 10.3314/jjmm.40.9 9929576

[B23] Nenoff P. Handrick W. Krüger C. Vissiennon T. Wichmann K. Gr?Ser Y.. (2012). [Dermatomycoses Due to Pets and Farm Animals: Neglected Infections]? Hautarzt 63, 848–858. doi: 10.1007/s00105-012-2379-y 23114507

[B24] North M. Steffen J. Loguinov A. V. Zimmerman G. R. Vulpe C. D. Eide D. J. (2012). Genome-Wide Functional Profiling Identifies Genes and Processes Important for Zinc-Limited Growth of Saccharomyces Cerevisiae. PloS Genet. 8, e1002699. doi: 10.1371/journal.pgen.1002699 22685415 PMC3369956

[B25] Petrucelli M. F. Peronni K. Sanches P. R. (2018). Dual RNA-Seq Analysis of Trichophyton Rubrum and HaCat Keratinocyte Co-Culture Highlights Important Genes for Fungal-Host Interaction. Genes (Basel) 9 (7), 362. doi: 10.3390/genes9070362 PMC6070946 30029541

[B26] Roberts A. (2011). Improving RNA-Seq Expression Estimates by Correcting for Fragment Bias. Genome Biol. 12, R22. doi: 10.1186/gb-2011-12-3-r22 21410973 PMC3129672

[B27] Satoru Y. Kumiko S. S. Aiko H. Tomoyuki S. Koki K. Emi S.. (2007). Zinc is a Novel Intracellular Second Messenger. J. Cell Biol. 177, 637–645. doi: 10.1083/jcb.200702081 17502426 PMC2064209

[B28] Schneider Rde O. Fogaça Nde S. Kmetzsch L. Schrank A. Vainstein M. H. Staats C. C. (2012). Zap1 Regulates Zinc Homeostasis and Modulates Virulence in Cryptococcus Gattii. PloS One 7, e43773. doi: 10.1371/journal.pone.0043773 22916306 PMC3423376

[B29] Shi Y. Niu Q. Yu X. Jia X. Wang J. Lin D.. (2016). Assessment of the Function of SUB6 in the Pathogenic Dermatophyte Trichophyton Mentagrophytes. Med. Mycol. 54, 59–71. doi: 10.1093/mmy/myv071 26333355

[B30] Silva-Bailão M. G. Silva K. L. P. D. Anjos L. R. B. D. Lima P. D. S. Teixeira M. D. M. Soares C. M. D. A.. (2017). Mechanisms of Copper and Zinc Homeostasis in Pathogenic Black Fungi. Fungal Biol. 122 (6), 526–537. doi: 10.1016/j.funbio.2017.12.002 29801797

[B31] Simon A. Paul Theodor P. Wolfgang H. (2015). HTSeq–a Python Framework to Work With High-Throughput Sequencing Data. Bioinformatics 31, 166–169. doi: 10.1093/bioinformatics/btu638 25260700 PMC4287950

[B32] Sleight S. C. Bartley B. A. Lieviant J. A. Sauro H. M. (2010). In-Fusion BioBrick Assembly and Re-Engineering. Nucleic Acids Res. 38, 2624–2636. doi: 10.1093/nar/gkq179 20385581 PMC2860134

[B33] Staats C. C. Kmetzsch L. Schrank A. Vainstein M. H. (2013). Fungal Zinc Metabolism and its Connections to Virulence. Front. Cell. Infect. Microbiol. 3, 65. doi: 10.3389/fcimb.2013.00065 24133658 PMC3796257

[B34] Stojanov I. M. Prodanov J. Z. Pušic´ I. M. Ratajac R. D. (2009). Dermatomycosis - a Potential Source of Zoonotic Infection in Cities. Zbornik Matice Srpske Za Prirodne Nauke 116, 275–280. doi: 10.2298/ZMSPN0916275S

[B35] Takeda K. Anzawa K. Mochizuki T. Yamada S. Kobayashi H. Kimura S. (2018). Infant Case of Tinea Faciei Caused by Microsporum Canis. J. Dermatol. 45 (7), e187–e188. doi: 10.1111/1346-8138.14242 29411427

[B36] Thokala S. Bodiga V. L. Kudle M. R. Bodiga S. (2019). Comparative Response of Cardiomyocyte ZIPs and ZnTs to Extracellular Zinc and TPEN. Biol. Trace Elem. Res. 192, 297–307. doi: 10.1007/s12011-019-01671-0 30778755

[B37] Wilson D. Citiulo F. Hube B. (2012). Zinc Exploitation by Pathogenic Fungi. PloS Pathog. 8, e1003034. doi: 10.1371/journal.ppat.1003034 23308062 PMC3534374

[B38] Xiao W. He H. Tong Y. Cai M. Shi Y. Liu B.. (2018). Transcriptome Analysis of Trichophyton Mentagrophytes-Induced Rabbit (Oryctolagus Cuniculus) Dermatophytosis. Microb. Pathog. 114, 350–356. doi: 10.1016/j.micpath.2017.12.018 29225090

[B39] Yamada Y. Makimura K. Merhendi H. Ueda K. Nishiyama Y. Yamaguchi H.. (2002). Comparison of Different Methods for Extraction of Mitochondrial DNA From Human Pathogenic Yeasts. JPN. J. Infect. Dis. 55, 122–125. 12403909

[B40] Yamada T. Makimura K. Satoh K. Umeda Y. Ishihara Y. Abe S. (2009). Agrobacterium Tumefaciens-Mediated Transformation of the Dermatophyte, Trichophyton Mentagrophytes: An Efficient Tool for Gene Transfer. Med. Mycol. 47, 485–494. doi: 10.1080/13693780802322240 18951290

[B41] Young M. D. Wakefield M. J. Smyth G. K. Alicia O. (2010). Gene Ontology Analysis for RNA-Seq: Accounting for Selection Bias. Genome Biol. 11, R14–R14. doi: 10.1186/gb-2010-11-2-r14 20132535 PMC2872874

[B42] Yu Z. Yu Z. (2019). Zinc Chelator TPEN Induces Pancreatic Cancer Cell Death Through Causing Oxidative Stress and Inhibiting Cell Autophagy. J. Cell Physiol. 234, 20648–20661. doi: 10.1002/jcp.28670 31054150

[B43] Zeilinger S. (2004). Gene Disruption in Trichoderma Atroviride *via* Agrobacterium-Mediated Transformation. Curr. Genet. 45, 54–60. doi: 10.1007/s00294-003-0454-8 14586554

[B44] Zhang X. Dai P. Gao Y. Gong X. Cui H. Jin Y.. (2017). Transcriptome Sequencing and Analysis of Zinc-Uptake-Related Genes in Trichophyton Mentagrophytes. BMC Genomics 18, 888. doi: 10.1186/s12864-017-4284-3 29157209 PMC5697147

[B45] Zhang X. Wang Y. Chi W. Shi Y. Chen S. Lin D.. (2014). Metalloprotease Genes of Trichophyton Mentagrophytes Are Important for Pathogenicity. Med. Mycol. 52, 36–45. doi: 10.3109/13693786.2013.811552 23859078

