# Supplementary material for: RNA-Seq Analysis of the Effect of Zinc Deficiency on Microsporum canis, ZafA Gene Is Important for Growth and Pathogenicity
Source: Front Cell Infect Microbiol. 2021 Sep 16;11:727665. doi: 10.3389/fcimb.2021.727665 (PMC8481874; doi:10.3389/fcimb.2021.727665)
Supplement: Supplementary Material 1 — The concentration, purity and integrity of RNA. [file DataSheet_1.zip › Supplementary Material 1.docx]

| Sample ID | Species | O.D. 260/280 | O.D. 260/230 | RIN | Conc. (ng/µL) | Amount (µg) | QC Evaluation |
| --- | --- | --- | --- | --- | --- | --- | --- |
| NORM_1 | *M．Canis* | 2.06 | 1.87 | 9.00 | 252 ng/uL | 7.22 | Qualified |
| NORM_2 | *M．Canis* | 2.03 | 1.86 | 8.90 | 286 ng/uL | 6.31 | Qualified |
| NORM_3 | *M．Canis* | 2.11 | 1.89 | 9.10 | 271 ng/uL | 6.82 | Qualified |
| Zn200_1 | *M．Canis* | 2.04 | 1.88 | 8.80 | 264 ng/uL | 6.67 | Qualified |
| Zn200_2 | *M．Canis* | 2.06 | 1.87 | 8.90 | 272 ng/uL | 7.36 | Qualified |
| Zn200_3 | *M．Canis* | 2.06 | 1.88 | 8.90 | 265 ng/uL | 6.61 | Qualified |
| Zn800_1 | *M．Canis* | 2.10 | 1.92 | 9.10 | 279 ng/uL | 7.01 | Qualified |
| Zn800_2 | *M．Canis* | 2.05 | 1.89 | 9.00 | 265 ng/uL | 6.98 | Qualified |
| Zn800_3 | *M．Canis* | 2.09 | 1.90 | 9.10 | 251 ng/uL | 6.39 | Qualified |

**The concentration, purity and integrity of RNA**
