# Supplementary material for: RNA-Seq Analysis of the Effect of Zinc Deficiency on Microsporum canis, ZafA Gene Is Important for Growth and Pathogenicity
Source: Front Cell Infect Microbiol. 2021 Sep 16;11:727665. doi: 10.3389/fcimb.2021.727665 (PMC8481874; doi:10.3389/fcimb.2021.727665)
Supplement: Supplementary Material 1 — The concentration, purity and integrity of RNA. [file DataSheet_1.zip › Supplementary Material 2.docx]

**Transcriptome sequencing quality**

| Type | NORM_1 | NORM_2 | NORM_3 | Zn200_1 | Zn200_2 | Zn200_3 | Zn800_1 | Zn800_2 | Zn800_3 |
| --- | --- | --- | --- | --- | --- | --- | --- | --- | --- |
| Raw Reads | 43800592 | 37714340 | 37312444 | 56423084 | 74925758 | 56345050 | 90812668 | 80820458 | 69708020 |
| Clean Reads | 43106926 | 37521190 | 37016674 | 56114678 | 74413850 | 55946808 | 90299722 | 80355972 | 69282180 |
| Clean Reads Ratio(%) | 98.42 | 99.49 | 99.21 | 99.45 | 99.32 | 99.29 | 99.44 | 99.43 | 99.39 |
| GC(%) | 48.37 | 49.94 | 49.97 | 51.34 | 51.12 | 51.45 | 50.92 | 51.05 | 51.34 |
| Q20(%) | 98.29 | 97.7 | 97.9 | 98.29 | 98.12 | 98.19 | 98.03 | 98.04 | 98.03 |
| Q30(%) | 94.84 | 93.66 | 94.28 | 94.8 | 94.41 | 94.56 | 94.19 | 94.17 | 94.13 |

**The statistics of reads correspond to genomes**

|  | Total_reads | Total_mapped_reads | Unique_mapped_reads | Reads_mapped_in_paired |
| --- | --- | --- | --- | --- |
| NORM_1 | 43106926 | 9.27% | 6.35% | 7.42% |
| NORM_2 | 37521190 | 93.34% | 90.19% | 90.26% |
| NORM_3 | 37016674 | 92.85% | 90.69% | 89.55% |
| Zn200_1 | 56114678 | 96.17% | 91.38% | 94.07% |
| Zn200_2 | 74413850 | 96.02% | 91.09% | 93.68% |
| Zn200_3 | 55946808 | 95.34% | 92.69% | 92.84% |
| Zn800_1 | 90299722 | 96.44% | 92.99% | 94.20% |
| Zn800_2 | 80355972 | 96.56% | 92.87% | 94.51% |
| Zn800_3 | 69282180 | 96.04% | 90.84% | 93.81% |
