# Supplementary material for: RNA-Seq Analysis of the Effect of Zinc Deficiency on Microsporum canis, ZafA Gene Is Important for Growth and Pathogenicity
Source: Front Cell Infect Microbiol. 2021 Sep 16;11:727665. doi: 10.3389/fcimb.2021.727665 (PMC8481874; doi:10.3389/fcimb.2021.727665)
Supplement: Supplementary Material 1 — The concentration, purity and integrity of RNA. [file DataSheet_1.zip › Supplementary Material 6.docx]

**NORM-vs-Zn200**

| Type | GoTerm | GeneNum | p-value |
| --- | --- | --- | --- |
| BP | syncytium formation by plasma membrane fusion | 1 | 0.00 |
| BP | protein polyubiquitination | 1 | 0.00 |
| BP | telomere maintenance | 1 | 0.00 |
| BP | mRNA splicing, via spliceosome | 1 | 0.00 |
| BP | cell wall mannoprotein biosynthetic process | 2 | 0.00 |
| BP | positive regulation of transcription by galactose | 1 | 0.00 |
| BP | sporocarp development involved in sexual reproduction | 2 | 0.00 |
| BP | maturation of LSU-rRNA from tricistronic rRNA transcript (SSU-rRNA, 5.8S rRNA, LSU-rRNA) | 1 | 0.00 |
| BP | maturation of 5.8S rRNA from tricistronic rRNA transcript (SSU-rRNA, 5.8S rRNA, LSU-rRNA) | 1 | 0.00 |
| BP | actin cortical patch assembly | 1 | 0.00 |
| BP | protein deneddylation | 1 | 0.00 |
| BP | exonucleolytic trimming to generate mature 3'-end of 5.8S rRNA from tricistronic rRNA transcript (SS | 1 | 0.00 |
| BP | cell morphogenesis | 1 | 0.00 |
| BP | positive regulation of transcription from RNA polymerase II promoter by galactose | 1 | 0.00 |
| BP | ribosomal large subunit assembly | 1 | 0.00 |
| BP | negative regulation of transcription from RNA polymerase II promoter | 2 | 0.00 |
| BP | barrier septum assembly | 2 | 0.00 |
| BP | sulfate assimilation | 1 | 0.00 |
| CC | nuclear chromatin | 3 | 0.00 |
| CC | alpha-1,6-mannosyltransferase complex | 1 | 0.00 |
| CC | Golgi membrane | 1 | 0.00 |
| CC | kinetochore | 3 | 0.00 |
| CC | fungal-type vacuole membrane | 3 | 0.00 |
| CC | incipient cellular bud site | 1 | 0.00 |
| CC | fungal-type vacuole | 2 | 0.00 |
| MF | magnesium ion binding | 2 | 0.00 |
| MF | nucleotide binding | 16 | 0.00 |
| MF | alpha-1,6-mannosyltransferase activity | 1 | 0.00 |
| MF | 3'-5'-exoribonuclease activity | 1 | 0.00 |
| MF | second spliceosomal transesterification activity | 1 | 0.00 |

**NORM-vs-Zn800**

| Type | GoTerm | GeneNum | p-value |
| --- | --- | --- | --- |
| BP | protein deneddylation | 1 | 0.00 |
| BP | telomere maintenance | 1 | 0.00 |
| BP | mitochondrion inheritance | 1 | 0.00 |
| BP | actin cortical patch assembly | 1 | 0.00 |
| BP | negative regulation of transcription from RNA polymerase II promoter | 2 | 0.00 |
| BP | ribosomal large subunit assembly | 1 | 0.00 |
| BP | cell wall mannoprotein biosynthetic process | 3 | 0.00 |
| BP | spliceosomal snRNP assembly | 1 | 0.00 |
| BP | positive regulation of transcription by galactose | 1 | 0.00 |
| BP | sulfate assimilation | 1 | 0.00 |
| BP | protein polyubiquitination | 1 | 0.00 |
| BP | maturation of 5.8S rRNA from tricistronic rRNA transcript (SSU-rRNA, 5.8S rRNA, LSU-rRNA) | 1 | 0.00 |
| BP | mRNA splicing, via spliceosome | 2 | 0.00 |
| BP | maturation of LSU-rRNA from tricistronic rRNA transcript (SSU-rRNA, 5.8S rRNA, LSU-rRNA) | 1 | 0.00 |
| CC | fungal-type vacuole membrane | 4 | 0.00 |
| CC | kinetochore | 3 | 0.00 |
| CC | extrinsic component of vacuolar membrane | 1 | 0.00 |
| CC | Golgi membrane | 2 | 0.00 |
| CC | fungal-type vacuole | 4 | 0.00 |
| CC | proteasome complex | 1 | 0.00 |
| CC | incipient cellular bud site | 1 | 0.00 |
| CC | protein storage vacuole | 1 | 0.00 |
| CC | contractile vacuole | 1 | 0.00 |
| CC | nuclear chromatin | 3 | 0.00 |
| CC | alpha-1,6-mannosyltransferase complex | 2 | 0.00 |
| MF | alpha-1,6-mannosyltransferase activity | 2 | 0.00 |
| MF | adenyl-nucleotide exchange factor activity | 1 | 0.00 |
| MF | magnesium ion binding | 1 | 0.00 |
| MF | nucleotide binding | 17 | 0.00 |
| MF | second spliceosomal transesterification activity | 1 | 0.00 |

**Zn800-vs-Zn200**

| Type | GoTerm | GeneNum | p-value |
| --- | --- | --- | --- |
| BP | protein folding in endoplasmic reticulum | 1 | 0.00 |
| BP | transmembrane transport | 1 | 0.00 |
| BP | oxidation-reduction process | 1 | 0.00 |
| CC | integral component of membrane | 1 | 0.00 |
| CC | integral component of plasma membrane | 1 | 0.00 |
| CC | endoplasmic reticulum membrane | 1 | 0.00 |
| CC | endoplasmic reticulum | 1 | 0.00 |
| MF | oxidoreductase activity, acting on a sulfur group of donors, disulfide as acceptor | 1 | 0.00 |
| MF | flavin adenine dinucleotide binding | 1 | 0.00 |
| MF | thiol oxidase activity | 1 | 0.00 |
| MF | transmembrane transporter activity | 1 | 0.00 |
| MF | protein disulfide isomerase activity | 1 | 0.00 |
| MF | protein disulfide oxidoreductase activity | 1 | 0.00 |
