# Supplementary material for: RNA-Seq Analysis of the Effect of Zinc Deficiency on Microsporum canis, ZafA Gene Is Important for Growth and Pathogenicity
Source: Front Cell Infect Microbiol. 2021 Sep 16;11:727665. doi: 10.3389/fcimb.2021.727665 (PMC8481874; doi:10.3389/fcimb.2021.727665)
Supplement: Supplementary Material 1 — The concentration, purity and integrity of RNA. [file DataSheet_1.zip › Supplementary Material 7.docx]

**NORM-vs-Zn200**

| Type | GoTerm | Up | Down | p-value |
| --- | --- | --- | --- | --- |
| Biological Process | positive regulation of transcription from RNA polymerase II promoter by galactose | 1 | 0 | 0.00 |
| Biological Process | protein polyubiquitination | 1 | 0 | 0.00 |
| Biological Process | sulfate assimilation | 1 | 0 | 0.00 |
| Biological Process | protein deneddylation | 0 | 1 | 0.00 |
| Biological Process | actin cortical patch assembly | 1 | 0 | 0.00 |
| Biological Process | ribosomal large subunit assembly | 0 | 1 | 0.00 |
| Biological Process | mRNA splicing, via spliceosome | 0 | 1 | 0.00 |
| Biological Process | negative regulation of transcription from RNA polymerase II promoter | 1 | 1 | 0.00 |
| Biological Process | cell wall mannoprotein biosynthetic process | 2 | 0 | 0.00 |
| Biological Process | positive regulation of transcription by galactose | 0 | 1 | 0.00 |
| Cellular Component | nuclear chromatin | 2 | 1 | 0.00 |
| Cellular Component | Golgi membrane | 1 | 0 | 0.00 |
| Cellular Component | kinetochore | 3 | 0 | 0.00 |
| Cellular Component | fungal-type vacuole membrane | 2 | 1 | 0.00 |
| Cellular Component | fungal-type vacuole | 2 | 0 | 0.00 |
| Cellular Component | nucleus | 32 | 17 | 0.00 |
| Cellular Component | alpha-1,6-mannosyltransferase complex | 1 | 0 | 0.00 |
| Cellular Component | barrier septum | 2 | 0 | 0.00 |
| Cellular Component | extracellular region | 1 | 7 | 0.00 |
| Cellular Component | incipient cellular bud site | 1 | 0 | 0.00 |
| Molecular Function | sequence-specific DNA binding RNA polymerase II transcription factor activity | 9 | 0 | 0.00 |
| Molecular Function | second spliceosomal transesterification activity | 0 | 1 | 0.00 |
| Molecular Function | 3'-5'-exoribonuclease activity | 1 | 0 | 0.00 |
| Molecular Function | alpha-1,6-mannosyltransferase activity | 1 | 0 | 0.00 |
| Molecular Function | RNA polymerase II core promoter sequence-specific DNA binding | 0 | 1 | 0.00 |
| Molecular Function | magnesium ion binding | 2 | 0 | 0.00 |
| Molecular Function | RNA polymerase I activity | 0 | 1 | 0.00 |
| Molecular Function | mitochondrial RNA polymerase regulatory region DNA binding | 1 | 0 | 0.00 |
| Molecular Function | RNA polymerase II core promoter proximal region sequence-specific DNA binding | 4 | 0 | 0.00 |
| Molecular Function | nucleotide binding | 8 | 8 | 0.00 |

**NORM-vs-Zn800**

| Type | GoTerm | Up | Down | p-value |
| --- | --- | --- | --- | --- |
| Biological Process | mRNA splicing, via spliceosome | 0 | 2 | 0.00 |
| Biological Process | sulfate assimilation | 1 | 0 | 0.00 |
| Biological Process | protein polyubiquitination | 1 | 0 | 0.00 |
| Biological Process | cell wall mannoprotein biosynthetic process | 3 | 0 | 0.00 |
| Biological Process | spliceosomal snRNP assembly | 0 | 1 | 0.00 |
| Biological Process | negative regulation of transcription from RNA polymerase II promoter | 1 | 1 | 0.00 |
| Biological Process | ribosomal large subunit assembly | 0 | 1 | 0.00 |
| Biological Process | mitochondrion inheritance | 1 | 0 | 0.00 |
| Biological Process | actin cortical patch assembly | 1 | 0 | 0.00 |
| Biological Process | protein deneddylation | 0 | 1 | 0.00 |
| Cellular Component | fungal-type vacuole membrane | 2 | 2 | 0.00 |
| Cellular Component | kinetochore | 3 | 0 | 0.00 |
| Cellular Component | extrinsic component of vacuolar membrane | 1 | 0 | 0.00 |
| Cellular Component | Golgi membrane | 2 | 0 | 0.00 |
| Cellular Component | fungal-type vacuole | 1 | 3 | 0.00 |
| Cellular Component | proteasome complex | 0 | 1 | 0.00 |
| Cellular Component | incipient cellular bud site | 1 | 0 | 0.00 |
| Cellular Component | protein storage vacuole | 1 | 0 | 0.00 |
| Cellular Component | contractile vacuole | 0 | 1 | 0.00 |
| Cellular Component | alpha-1,6-mannosyltransferase complex | 2 | 0 | 0.00 |
| Molecular Function | alpha-1,6-mannosyltransferase activity | 2 | 0 | 0.00 |
| Molecular Function | mitochondrial RNA polymerase regulatory region DNA binding | 1 | 0 | 0.00 |
| Molecular Function | adenyl-nucleotide exchange factor activity | 1 | 0 | 0.00 |
| Molecular Function | RNA polymerase I activity | 0 | 1 | 0.00 |
| Molecular Function | nucleotide binding | 8 | 9 | 0.00 |
| Molecular Function | sequence-specific DNA binding RNA polymerase II transcription factor activity | 8 | 0 | 0.00 |
| Molecular Function | RNA polymerase II core promoter proximal region sequence-specific DNA binding | 3 | 0 | 0.00 |
| Molecular Function | magnesium ion binding | 1 | 0 | 0.00 |
| Molecular Function | second spliceosomal transesterification activity | 0 | 1 | 0.00 |
| Molecular Function | RNA polymerase II core promoter sequence-specific DNA binding | 0 | 1 | 0.00 |

**Zn800-vs-Zn200**

| Type | GoTerm | Up | Down | p-value |
| --- | --- | --- | --- | --- |
| Biological Process | transmembrane transport | 1 | 0 | 0.00 |
| Biological Process | oxidation-reduction process | 0 | 1 | 0.00 |
| Biological Process | protein folding in endoplasmic reticulum | 0 | 1 | 0.00 |
| Cellular Component | endoplasmic reticulum membrane | 0 | 1 | 0.00 |
| Cellular Component | endoplasmic reticulum | 1 | 0 | 0.00 |
| Cellular Component | integral component of membrane | 1 | 0 | 0.00 |
| Cellular Component | integral component of plasma membrane | 1 | 0 | 0.00 |
| Molecular Function | flavin adenine dinucleotide binding | 0 | 1 | 0.00 |
| Molecular Function | transmembrane transporter activity | 1 | 0 | 0.00 |
| Molecular Function | thiol oxidase activity | 0 | 1 | 0.00 |
| Molecular Function | protein disulfide isomerase activity | 0 | 1 | 0.00 |
| Molecular Function | protein disulfide oxidoreductase activity | 0 | 1 | 0.00 |
| Molecular Function | oxidoreductase activity, acting on a sulfur group of donors, disulfide as acceptor | 0 | 1 | 0.00 |
