# Supplementary material for: RNA-Seq Analysis of the Effect of Zinc Deficiency on Microsporum canis, ZafA Gene Is Important for Growth and Pathogenicity
Source: Front Cell Infect Microbiol. 2021 Sep 16;11:727665. doi: 10.3389/fcimb.2021.727665 (PMC8481874; doi:10.3389/fcimb.2021.727665)
Supplement: Supplementary Material 1 — The concentration, purity and integrity of RNA. [file DataSheet_1.zip › Supplementary Material 14.docx]

| Gene id | Log2fold | P-value | Q-value | Diff | Gene Description |
| --- | --- | --- | --- | --- | --- |
| MCYG_04541 | 4.73376469 | 0.00 | 0.00 | Up | Hypothetical Protein |
| MCYG_02505 | 3.418847262 | 0.00 | 0.00 | Up | Hypothetical Protein |
| MCYG_00683 | 2.72646729 | 0.00 | 0.00 | Up | Hypothetical Protein |
| MCYG_04543 | 5.010837092 | 0.00 | 0.00 | Up | Tetracycline Efflux Protein |
| MCYG_07519 | 3.076429617 | 0.00 | 0.00 | Up | Keratin-Associated Protein |
| MCYG_03529 | 4.431672282 | 0.00 | 0.00 | Up | Hypothetical Protein |
| MCYG_00714 | 2.729017694 | 0.00 | 0.00 | Up | Meiotically Up-Regulated Gene 80 Protein |
| MCYG_02705 | 2.353977528 | 0.00 | 0.00 | Up | Oxalate Decarboxylase |
| MCYG_02497 | 3.194045383 | 0.00 | 0.00 | Up | G1/S-Specific Cyclin CLN1 |
| MCYG_05216 | 3.55212156 | 0.00 | 0.00 | Up | ABC Multidrug Transporter Mdr1 |
| MCYG_06825 | 3.188360087 | 0.00 | 0.00 | Up | Pumilio-Family RNA Binding Protein |
| MCYG_01022 | 3.339619209 | 0.00 | 0.00 | Up | Hypothetical Protein |
| MCYG_01569 | 3.66017886 | 0.00 | 0.00 | Up | Calcium P-Type Atpase |
| MCYG_03941 | 2.098904067 | 0.00 | 0.00 | Up | Sphingosine N-Acyltransferase Lac1 |
| MCYG_05608 | 4.949203314 | 0.00 | 0.00 | Up | Zinc/Iron Transporter Protein |
| MCYG_03992 | 3.132985052 | 0.00 | 0.00 | Up | Hypothetical Protein |
| MCYG_03534 | 3.166190661 | 0.00 | 0.00 | Up | RNA-Binding Protein Nrd1 |
| MCYG_07841 | 5.215190964 | 0.00 | 0.00 | Up | Tetracycline Efflux Protein |
| MCYG_02984 | 2.625002427 | 0.00 | 0.00 | Up | Hypothetical Protein |
| MCYG_04688 | 3.078558354 | 0.00 | 0.00 | Up | DNA Repair Protein |

**20 DEGs with significantly increased expression in *M. Canis* under low zinc concentration culture**
