# Supplementary material for: RNA-Seq Analysis of the Effect of Zinc Deficiency on Microsporum canis, ZafA Gene Is Important for Growth and Pathogenicity
Source: Front Cell Infect Microbiol. 2021 Sep 16;11:727665. doi: 10.3389/fcimb.2021.727665 (PMC8481874; doi:10.3389/fcimb.2021.727665)
Supplement: Supplementary Material 1 — The concentration, purity and integrity of RNA. [file DataSheet_1.zip › Supplementary Material 15.docx]

The sequences of primers

| The name of primers | The sequences of primers |
| --- | --- |
| *β-actin-F1* | GCTTCTAGGCGGACTGTTAC (20 bp) |
| *β-actin-R1* | CCATGCCAATGTTGTCTCTT (20 bp) |
| *MCYG_04486-F1* | GCATAGCAGCACACCATCTC (20 bp) |
| *MCYG_04486-R1* | CACTTTCCGTTTGCACAGGA (20 bp) |
| *MCYG_02504-F1* | TGACTACAATGGCCAGCTCA (20 bp) |
| *MCYG_02504-R1* | TTCGAAGGTGAGTCTGGGTC (20 bp) |
| *MCYG_06235-F1* | TCAAAGCTCCAGGGCCATTA (20 bp) |
| *MCYG_06235-R1* | TCGAAATTGCAACCAGGGTG (20 bp) |
| *MCYG_02286 -F1* | TTGAGTTCCACTGGTACGCT (20 bp) |
| *MCYG_02286 -R1* | ATGGTGGGGATTTGGCAGTA (20 bp) |
| *MCYG_07837-F1* | CATACATTCGCACTCACGCA (20 bp) |
| *MCYG_07837-R1* | TCAGCCGTCTCAGTATCGAC (20 bp) |
| *MCYG_00110-F1* | CGTGGTTAATCTGCCGTCTG (20 bp) |
| *MCYG_00110-R1* | TGGTGCTTGAGGGTACTGAG (20 bp) |
| *MCYG_04543-F1* | CACCAGCGACTGTTCAACAA (20 bp) |
| *MCYG_04543-R1* | GAATGAACCTGTGAGCGTCC (20 bp) |
| *MCYG_05608-F1* | GACCCATGACTACCCACACA (20 bp) |
| *MCYG_05608-R1* | CACCCCGACTTCTTCTCCAT (20 bp) |
| *MCYG_07841-F1* | GAGAATCCGCTCCGTCCTTA (20 bp) |
| *MCYG_07841-R1* | ACCGTCCTCTTCCTAGACCT (20 bp) |
| *MCYG_01475-F1* | CTCCTACGGTGGTGCAAATG (20 bp) |
| *MCYG_01475-R1* | TCCATGGAAAACAACGGCTG (20 bp) |
| *MCYG_04785-F1* | CGGCCATCAGAGTCTCATCT (20 bp) |
| *MCYG_04785-R1* | AATGTCCTTGCATGCGTTGT (20 bp) |
| *MCYG_08408-F1* | ACCCTCTCCGCATCCATAAG (20 bp) |
| *MCYG_08408-R1* | CGTGGATGATGGTAGTCGGA (20 bp) |
| *MCYG_04541-F1* | TGTGAATGACTCTCGGATTA (20 bp) |
| *MCYG_04541-R1* | GATGTGCTCGGAAGGTAG (18 bp) |
| *MCYG_02505-F1* | AGAACTACCACGCCATTAC (19 bp) |
| *MCYG_02505-R1* | TTACTTAGCACGCCACTTAT (20 bp) |
| *MCYG_00683-F1* | GACGACAGCAACATCAAC (18 bp) |
| *MCYG_00683-R1* | TGGACAGGTAAGGCAGAT (18 bp) |
| *MCYG_04543-F1* | GTGATGATGCCGTAGGAAT (19 bp) |
| *MCYG_04543-R1* | TGCGAATTACTGAGCCATAT (20 bp) |
| *MCYG_07519-F1* | TAGCGGTTACAGAGAATGC (19 bp) |
| *MCYG_07519-R1* | TGAAGACGATGGAGAGGAT (19 bp) |
| *MCYG_03529-F1* | TCACCATCACCATCATCAC (19 bp) |
| *MCYG_03529-R1* | CAGCGAACCACTCCATAG (18 bp) |
| *MCYG_00714-F1* | GCAACAACAGCAACAACA (18 bp) |
| *MCYG_00714-R1* | AGAGACCGAGAAGGATGG (18 bp) |
| *MCYG_02705-F1* | CTAAGGAGGTATTGGCTAAGA (21 bp) |
| *MCYG_02705-R1* | AGTTCAAGGTAGACGAGTTC (20 bp) |
| *MCYG_02497-F1* | GACGAGTATCAGGAAGACAT (20 bp) |
| *MCYG_02497-R1* | ATAAGGTGGCAGGTAGCA (18 bp) |
| *MCYG_05216-F1* | TCATCCAAGAAGGCATCAC (19 bp) |
| *MCYG_05216-R1* | AGAAGCAGAGACCGTAGG (18 bp) |
| *MCYG_06825-F1* | TCACGAAGTAATGGCACAA (19 bp) |
| *MCYG_06825-R1* | TCAGAGACAGCAGCAGAT (18 bp) |
| *MCYG_01022-F1* | CGCTATGGATTCTACCTTGT (20 bp) |
| *MCYG_01022-R1* | GTCTATGTTCGGCTCTTGT (19 bp) |
| *MCYG_01569-F1* | CCGTCAGCACTACATCTAA (19 bp) |
| *MCYG_01569-R1* | GGTCGTTCCTCACATCAA (18 bp) |
| *MCYG_03941-F1* | TGTGATGAAGCAGACGAAT (19 bp) |
| *MCYG_03941-R1* | GATGTGGAGGTTGAGGTAAT (20 bp) |
| *MCYG_05608-F1* | CGCTACTGCTCAGACTAC (18 bp) |
| *MCYG_05608-R1* | CGGTAAGGTATGTTGTATTCTC (22 bp) |
| *MCYG_03992-F1* | TTAGTTACGCCGAAGGAAT (19 bp) |
| *MCYG_03992-R1* | GTATCTGATGATGCTGTAGAC (21 bp) |
| *MCYG_03534-F1* | CCGTCACTGGTTCTTCTG (18 bp) |
| *MCYG_03534-R1* | CACTCCGTTGCTACTTCC (18 bp) |
| *MCYG_07841-F1* | GTGCGGAACAATCTTGAATA (20 bp) |
| *MCYG_07841-R1* | TGGTAGGCTGGAATAAGGA (19 bp) |
| *MCYG_02984-F1* | CGCAGCAATAGCAATAGC (18 bp) |
| *MCYG_02984-R1* | AGGAGGAAGAAGCAGTGT (18 bp) |
| *MCYG_04688-F1* | AGGTTCTGCTCTTCATTCAA (20 bp) |
| *MCYG_04688-R1* | GTGTCTCTAATCTCGCTCTT (20 bp) |
| *ZrtaⅠF* | gtaccgggccccccctcgagTCACTGCTTCCACCTGCATTAC (42 bp) |
| *ZrtaⅠR* | cattctccttcgcttTTGCCGTCTGCGTCTAGATTTG (37 bp) |
| *HphF* | agacgcagacggcaaAAGCGAAGGAGAATGTGAAGCC (37 bp) |
| *HphR* | ctgagaaagcgggttGCTGTATCTGGAAGAGGTAAAC (37 bp) |
| *ZrtaⅡF* | tcttccagatacagcAACCCGCTTTCTCAGACCAGTT (37 bp) |
| *ZrtaⅡR* | aggaattcgatatcaagcttCCGCTTATGACTCCACGGATAT (42 bp) |
| *hphF* | TGCTACATCCATACTCCATC (20 bp) |
| *hphR* | GTCTGCTGCTCCATACAA (18 bp) |
| *Zrtaq-F* | AATGGCGATTCAGGACTTAT (20 bp) |
| *Zrtaq-R* | ATGGATGGACGGATAGGAA (19 bp) |
| *Southern-F* | TTCTTTAGCCAGGGAGTA (18 bp) |
| *Southern-R* | GAAATGGTGGAGGTGTTA (18 bp) |

Underline base is the overlap region
